# Impact of Epicatechin on the Procoagulant Activities of Microparticles

**DOI:** 10.3390/nu12102935

**Published:** 2020-09-25

**Authors:** Thomas Sinegre, Dragan Milenkovic, Céline Bourgne, Dorian Teissandier, Yosra Nasri, Louis-Thomas Dannus, Christine Morand, Aurélien Lebreton

**Affiliations:** 1 UNH Unité de Nutrition Humaine, Institut National de Recherche pour l’Agriculture, l’Alimentation et l’Environnement, Université Clermont Auvergne, 63000 Clermont-Ferrand, France; dragan.milenkovic@inra.fr (D.M.); dteissandier@chu-clermontferrand.fr (D.T.); christine.morand@inrae.fr (C.M.); alebreton@chu-clermontferrand.fr (A.L.); 2 CHU Clermont-Ferrand, Service d’Hématologie Biologique, 63000 Clermont-Ferrand, France; cbourgne@chu-clermontferrand.fr (C.B.); ynasri@chu-clermontferrand.fr (Y.N.); ltdannus@chu-clermontferrand.fr (L.-T.D.)

**Keywords:** cardiovascular disease, coagulation, epicatechin, hemostasis, microparticles

## Abstract

Microparticles play a role in cardiovascular disease pathology. The flavanol-like epicatechin is increasingly considered due to its cardioprotective effects. The aim of this study was to investigate the impact of epicatechin on microparticle generation, phenotype and procoagulant properties. Plasma samples from 15 healthy subjects were incubated with increasing concentrations of epicatechin (1 to 100 μM). Then, the expression of glycoprotein IIb, phosphatidylserine (PS), glycoprotein Ib (GPIb) and P-selectin was assessed by flow cytometry analysis after (or not) platelet stimulation. Microparticle procoagulant activity was determined using Zymuphen^TM^ MP and Zymuphen^TM^ MP-TF for phospholipid and tissue factor content, and with thrombin generation (TG) assays for procoagulant function. Platelet microparticles that express GPIb (/µL) decreased from 20,743 ± 24,985 (vehicle) to 14,939 ± 14,333 (*p* = 0.6), 21,366 ± 16,949 (*p* = 0.9) and 15,425 ± 9953 (*p* < 0.05) in samples incubated with 1, 10 and 100 µM epicatechin, respectively. Microparticle concentration (nM PS) decreased from 5.6 ± 2.0 (vehicle) to 5.1 ± 2.2 (*p* = 0.5), 4.5 ± 1.5 (*p* < 0.05) and 4.7 ± 2.0 (*p* < 0.05) in samples incubated with 1, 10 and 100µM epicatechin, respectively. Epicatechin had no impact on tissue factor-positive microparticle concentration. Epicatechin decreased TG (endogenous thrombin potential, nM.min) from 586 ± 302 to 509 ± 226 (*p* = 0.3), 512 ± 270 (*p* = 0.3) and 445 ± 283 (*p* < 0.05). These findings indicate that epicatechin affects microparticle release, phenotype and procoagulant properties.

## 1. Introduction

Microparticles are small, anucleate vesicles ranging from 100 to 1000 nm in size. They are released by many cell types, including platelets, monocytes, red and endothelial cells by exocytosis from the cell membranes upon cell activation, stress or apoptosis [1]. Microparticles are delimited by a phospholipid bilayer and express proteins from the cell of origin [2]. Microparticles are detected in healthy subjects, and their release increases in various pathological conditions, such as cancer, diabetes, sepsis and cardiovascular diseases (CVD) [3,4,5].

Platelet microparticles (PMPs) represent the main fraction of circulating microparticles and are formed upon platelet activation, glycoprotein (Gp) IIb-IIIa signaling or after exposure to shear stress [6,7]. PMPs express Gp IIb-IIIa (CD41) and Gp Ib (CD42b), phospholipids (e.g., phosphatidylserine (PS) and phosphatidylcholine) and activation markers, such as P-selectin (CD62P) [8]. There are growing evidences for PMP physiological and pathological roles. For instance, PMP can interfere in cell-to-cell communication through various mechanisms, from the direct stimulation of target receptors to integration of surface receptors in a target cell. PMPs can also transfer their cytoplasmic content, including RNA [9], and modulate the adaptive immunity by stimulating the immune response [10]. PMPs also play a major role in pathological processes, such as infections, mainly through the transfer of receptors to recipient cells to allow virus entry, and in systemic diseases, such as rheumatoid arthritis, by amplification of the local inflammatory response [11]. PMPs also play a role in cancer development by favoring angiogenesis and tumor cell invasion, and can be predictive markers of metastasis formation and tumor aggressiveness [12,13,14]. Due to their high procoagulant activity, PMPs are a prognostic marker of choice in CVD [15,16]. Indeed, PMP concentration is increased following myocardial damage after acute myocardial infarction [17], particularly PMP sub-populations that express specific markers, such as P-selectin [18]. Large PMPs are associated with specific conditions, such as carotid atherosclerosis [19] and myocardial infarction, and also with the circulation of the thrombin-antithrombin complex and soluble CD40 ligand [20], the increase of which correlates with higher risk of cardiovascular events [21]. PMPs seem to play a key role in CVD due to the expression of phospholipids and procoagulant surface markers, and their capacity to recruit leukocytes, to transfer miRNAs and to promote inflammation [22,23,24].

Several studies demonstrated that a diet rich in polyphenols is inversely associated with CVD risk and mortality [25,26,27]. It has also been suggested that polyphenols influence the functions of platelets, one of the major players in the atherothrombotic process. Different polyphenols and especially flavonoids can modulate platelet activation and aggregation [28,29,30]. Epicatechin is a major representative of the flavanol subclass of flavonoids due to its abundance in highly consumed plant foods, such as cocoa, apples, tea and grapes [31,32]. A wealth of data from preclinical models and clinical trials indicates that epicatechin has cardioprotective effects by improving endothelial and platelet functions, reducing blood pressure and limiting atherosclerosis [33,34]. It has been reported that different polyphenols and plant food extracts can affect PMP formation [35,36,37,38]; however, to our knowledge, there is no study on the specific impact of epicatechin on PMPs. The aim of this study was to investigate in vitro epicatechin effect on PMP generation, phenotype and procoagulant properties by incubating human plasma with increasing concentrations of epicatechin.

## 2. Materials and Methods

### 2.1. Subjects

Fifteen healthy volunteers, 12 women and 3 men (mean age 30 years (21–42)) were enrolled. Exclusion criteria were history of bleeding and thromboembolism, ongoing antiplatelet drug or anticoagulant therapy and abnormal blood cell count, including thrombocytopenia <150 G/L and coagulation disorders fibrinogen <2.0 or >4.0 g/L, prothrombin time >15.5 s and activated partial thromboplastin time >39 s. Ethical approval was obtained from the local ethics committee (CPP Sud-Est VI, ref AU765).

### 2.2. Blood Sampling and Processing

Blood was collected by venipuncture in 0.109 M citrate tubes (Beckton Dickinson, le Pont de Claix, France) with 15 µg/mL of corn trypsin inhibitor to inhibit contact factor activation and after discarding the first few milliliters of blood. Platelet-rich plasma (PRP) was obtained by centrifugation at 200 g at room temperature for 10 min, without brake. Platelet-poor plasma (PPP) was prepared by double centrifugation (2200 g, 20 °C for 15 min) with an intermediate plasma decantation, according to the International Society on Thrombosis and Hemostasis (ISTH) guidelines [39]. PPP samples were stored at −80 °C until testing (less than 3 months), if necessary. Before the experiments, frozen plasma samples were thawed in a water bath at 37 °C for 5 min. Microparticle-rich plasma (MRP) was obtained from PPP centrifugation at 14,000 g at room temperature for 1 h. The microparticle-containing pellet was resuspended in 100 μL of the supernatant after centrifugation (and thus microparticle-poor) to concentrate microparticles by 5.0-fold.

(-)-Epicatechin stock solution (Extrasynthèse, Lyon, France; 12.5 mM in DMSO) was diluted with phosphate buffered saline (PBS) to 0.1, 1 and 10 mM working solutions that were then added to the plasma samples to reach the target final concentrations of 1, 10 and 100 µM with a constant 1/100 dilution. An equivalent volume of vehicle was added to samples without epicatechin. Plasma samples were incubated with epicatechin at 37 °C for 10 min.

#### Cytometry Analysis of Microparticles

**Figure A1:**
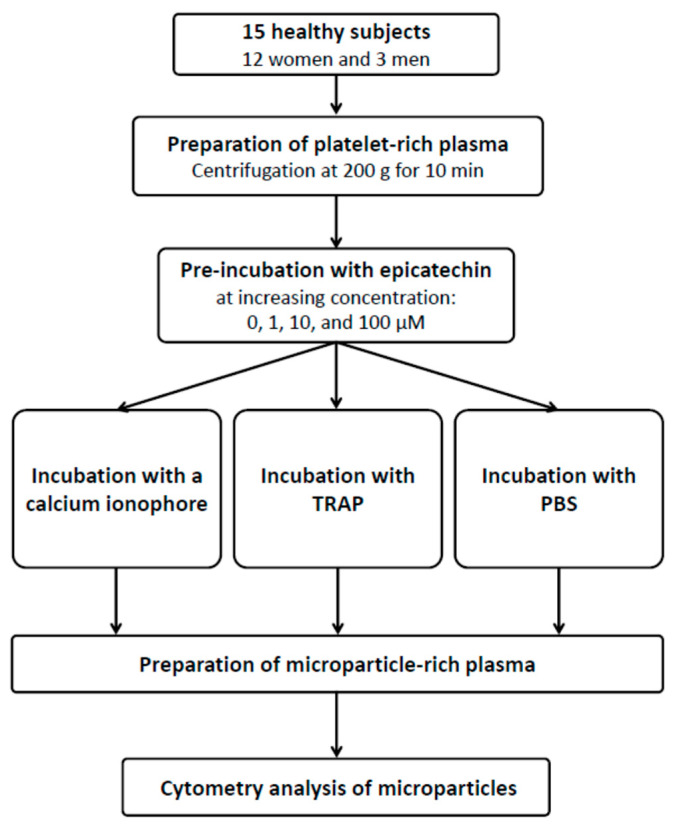
Study flow diagram describing the cytometry analysis of microparticles. TRAP: thrombin receptor activating peptide; PBS: phosphate buffered saline.

All cytometry analyses were performed with fresh samples. The impact of epicatechin on microparticles was determined in three conditions (all samples were pre-incubated with epicatechin): after incubation of PRP samples with PBS, to assess the direct role of epicatechin on microparticle production and their phospholipid and protein membrane composition (condition 1); after PRP incubation with platelet activators to simulate microparticle production (calcium ionophore A23187 (Sigma-Aldrich, Saint-Louis, MO, USA)) (condition 2) and with thrombin receptor activating peptide (TRAP; Roche, Mannheim, Germany) (condition 3), and to study epicatechin role in microparticle production and composition (Figure A1). Afterwards, samples were incubated with FITC-conjugated annexin-V (PS labeling), anti-CD41a-PE (glycoprotein IIb, clone HIP8), anti-CD42b-APC (glycoprotein Ib, clone HIP1) and anti-CD62P-BV421 (P-selectin, clone AK-4) antibodies (all from BD Biosciences) at room temperature in the dark for 20 min. Isotype controls (at the same concentration as the primary antibodies) were used as negative controls to differentiate non-specific background and specific antibody signals. Immediately after labeling, samples were resuspended in 250 µL of 0.20 µm-filtered annexin-V buffer and were analyzed on a BD FACS Canto II (BD Biosciences, Le Pont de Claix, France), equipped with three lasers (407, 488 and 633 nm wavelengths) and the BDFACS Diva software (v.8.0.1).

Flow cytometer performance tracking was performed daily using the BD cytometer setup and tracking beads (BD Biosciences). To ensure a limited background noise, filtered PBS (0.20 µm filter) was analyzed before each run at least for 10 min.

For each analysis, 100 µL of fresh PPP diluted at 1/100 or 1/200 was transferred to a TruCount tube (BD Biosciences) containing a lyophilized pellet that releases a known number of fluorescent beads to allow microparticle quantification.

Before each series of sample analysis, fluorescent Megamix-Plus SSC Beads (Biocytex, Marseille, France), a mix of fluorescent beads ranging from 0.1 to 1 µm, were used to define the gate consistent with the microparticle size, according to the manufacturer’s instructions. The Megamix-Plus by its standardized acquisition defines microparticles between 0.17 and 0.5 µm equivalent-SSC and allows discriminating between small and large microparticles (i.e., smaller and bigger than 0.22 µm-eq SSC). PMPs were characterized on the basis of the side scatter threshold defined using Megamix-Plus SSC Beads and labeling with CD41a, a constitutive platelet receptor.

### 2.3. Phospholipid-Induced Procoagulant Activity of Microparticles

Microparticle procoagulant activity was determined using Zymuphen^TM^ MP-ACTIVITY (Hyphen-biomed, Neuville, France), a functional immunological assay, according to the manufacturer’s protocol. Briefly, after pre-incubation with epicatechin, 5 µL of each PPP sample was incubated in a well of a microplate coated with annexin V that can bind to electronegative phospholipids at the microparticle surface. In the presence of calcium, factors (F) Xa and FVa, prothrombin are activated into thrombin in relation with microparticle exposure to phospholipids. Thrombin activity was measured by absorbance at 405 nm on a spectrophotometer (Spark, Tecan, Switzerland) following cleavage of a specific substrate. Plasma microparticle concentration was expressed in nM of PS equivalent.

### 2.4. Tissue factor (TF)-Induced Procoagulant Activity of Microparticles

Microparticle procoagulant activity was determined using Zymuphen^TM^ MP-TF (Hyphen-biomed), a functional immunological assay, according to the manufacturer’s protocol. Briefly, after pre-incubation with epicatechin, 20 µL of PPP was incubated in wells coated with an anti-TF monoclonal antibody. In the presence of calcium, FVIIa and FX, TF-positive microparticles form the TF-FVIIa complex and activate FX into FXa. TF-induced microparticle procoagulant activity was correlated with FXa activity on a specific substrate measured by absorbance at 405 nm on a spectrophotometer (Tecan) and was expressed in pg/mL.

### 2.5. Thrombin Generation Assays

Thrombin generation assays (TGA) were used in two experimental conditions to measure epicatechin effect on microparticle procoagulant activity by following the thrombin formation kinetics. First, MRP samples were incubated with increasing concentrations of epicatechin, as before, to evaluate the anticoagulant impact of epicatechin. Second, PRP samples were incubated with epicatechin before platelet stimulation by addition of 20 µM of the calcium ionophore A23187 (Sigma-Aldrich) that promotes platelet activation and apoptosis [40]. Then, MRPs were prepared as described above, and TGA were performed to evaluate the impact of epicatechin on the release of procoagulant microparticles from platelets.

TGA were performed using a modified Calibrated Automated Thrombogram method developed by Hemker [41], with a fluorometer (Fluoroscan Ascent, ThermoLab Systems, Franklin, TN, USA) equipped with a dispenser. Briefly, in 96-well plates (Immulon 2HB, Waltham, MA, USA), 30 µL of MRP samples was used as the source of phospholipids and TF. Then, 70 µL of a pool of normal plasma from 10 healthy donors, 20 µL of fluorogenic substrate and CaCl_2_ (FluCa-Kit^®^, Thrombinoscope BV) were added to the wells. In parallel, each sample was calibrated with Thrombin Calibrator^®^ (Stago, Asnières, France) and the same pool of normal plasma. The main parameter was the endogenous thrombin potential (ETP, area under the curve). All tests were performed in duplicate with a maximum difference <10% for ETP (nM.min) between curves. Raw data were analyzed using Thrombinoscope^TM^ V5 (Thrombinoscope BV, Maastricht, The Netherlands).

### 2.6. Statistical Analysis

Statistical analyses were performed with the Prism software, version 6 (GraphPad software, Inc., La Jolla, CA, USA). Tests were two-sided, with a type I error set at α = 0.05. Continuous data were presented as the mean ± standard deviation (SD). The statistical significance of differences between classes was determined with ANOVA, or the Friedman test when the ANOVA conditions were not met (normality and homoscedasticity verified with the Bartlett test), followed by the appropriate multiple comparison post-hoc tests (Tukey–Kramer or Dunn test, respectively).

## 3. Results

### 3.1. PMP Phenotype by Flow Cytometry

Pre-incubation with epicatechin did not have any effect on PMP concentration (PMP/µL) in PRP samples incubated with PBS (64,153 ± 45,388 for samples incubated with vehicle and 68,726 ± 41,966, 77,864 ± 49,161 and 59,841 ± 29,101 for samples incubated with 1 µM, 10 µM and 100 µM of epicatechin, respectively), with the calcium ionophore (537,613 ± 481,651 PMP/µL for samples incubated with vehicle and 683,474 ± 469,130, 551,204 ± 349,680 and 578,943 ± 375,997 for samples incubated with 1 µM, 10 µM and 100 µM of epicatechin, respectively) and with TRAP (100,870 ± 97,521 PMP/µL for samples incubated with vehicle and 117,215 ± 68,917, 101,101 ± 46,059 and 111,693 ± 76,321 for samples incubated with 1 µM, 10 µM and 100 µM of epicatechin, respectively). The concentrations of the two PMP populations (small and large) were not modified by pre-incubation with epicatechin, in all tested conditions. Of note, the inter-individual variability of PMP concentration was high, as indicated by the coefficient of variation of 70%.

On the other hand, epicatechin influenced the expression of CD62P at the PMP surface. When PRP was incubated with PBS, the percentage of CD62P-positive PMP decreased from 20.5 ± 13.7 in samples incubated with vehicle to 15.1 ± 10.4 (*p* < 0.05), 18.6 ± 11.5 (*p* = 0.34) and 15.3 ± 9.3 (*p* < 0.05) in samples incubated with 1 µM, 10 µM and 100 µM of epicatechin, respectively. The percentage of small and large CD62P-positive PMP also was modified: 13.4 ± 10.5 versus 7.8 ± 9.3 (*p* < 0.05), 9.3 ± 8.6 (*p* = 0.17), 7.4 ± 6.7 (*p* < 0.05) for small PMP, and 21.4 ± 14.3 versus 16.1 ± 9.5 (*p* < 0.05), 19.6 ± 12.3 (*p* = 0.16), 16.6 ± 9.7 (*p* < 0.05) for large PMP in samples incubated with vehicle versus 1 µM, 10 µM and 100 µM epicatechin, respectively. When PRP samples were incubated with the calcium ionophore or TRAP, the percentage of CD62P-positive PMP remained stable (31.4 ± 17.2 to 34.4 ± 11.9, and 13.0 ± 9.6 to 13.4 ± 9.5 for samples pre-incubated with vehicle and 100 µM epicatechin followed by calcium ionophore and TRAP, respectively), without any impact on the two sub-populations (small and large).

Epicatechin did not have any effect on the expression of PS (bound to annexin V), a driving force of the coagulation propagation phase. PS-positive PMP concentration (per µL) remained stable after incubation of PRP samples with PBS (51,136 ± 32,617 for samples incubated with vehicle and 57,889 ± 33,060, 64,977 ± 42,499 and 49,983 ± 25,245 for samples incubated with 1 µM, 10 µM and 100 µM epicatechin, respectively), with the calcium ionophore (471,828 ± 317,764 for samples incubated with vehicle and 571,121 ± 367,628 for samples incubated with 100 µM epicatechin) and with TRAP (84,045 ± 89,370 for samples incubated with vehicle and 81,023 ± 42,741 for samples incubated with 100 µM epicatechin). After incubation with the calcium ionophore and TRAP, PS-positive PMP concentration remained stable also in the two sub-populations.

**Figure 1:**
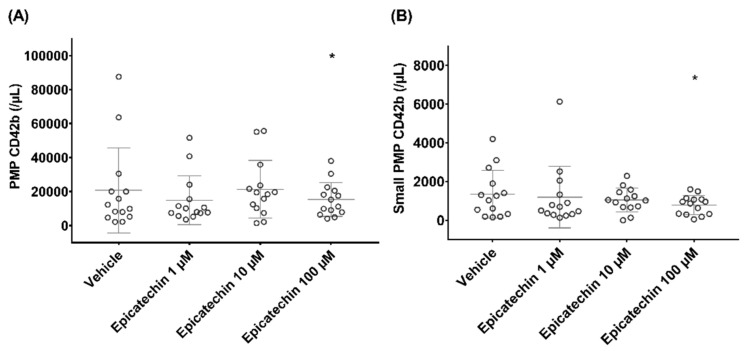
Impact of epicatechin on the concentration of platelet microparticles (PMPs) that express glycoprotein Ib. (**A**) Total PMP population. (**B**) Small PMP subpopulation. * Significant effect of intensity.

Epicatechin influenced the expression of CD42b (glycoprotein Ib) that participates in the coagulation process. After incubation with PBS, CD42b-positive PMP concentration (PMP/µL) decreased from 20743 ± 24985 in samples incubated with vehicle to 14,939 ± 14,333 (*p* = 0.6), 21,366 ± 16,949 (*p* = 0.9) and 15,425 ± 9953 (*p* < 0.05) in samples incubated with 1 µM, 10 µM and 100 µM epicatechin, respectively (Figure 1A). This decrease was mainly explained by the reduction in the concentration of CD42b-positive PMPs in the small subpopulation, from 1350 ± 1234 (in samples incubated with vehicle) to 783 ± 488 (*p* < 0.05) (in samples incubated with 100 µM epicatechin) (Figure 1B), while the large CD42b-positive PMP subpopulation remained stable.

**Figure 2:**
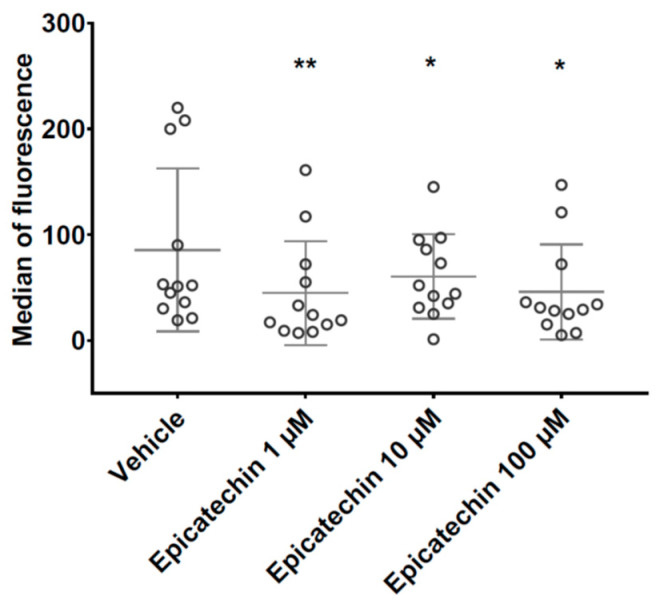
Impact of epicatechin on glycoprotein Ib expression at the surface of small PMPs. *, ** Significant effect of intensity.

After incubation with the calcium ionophore or TRAP, the concentration of CD42b-positive PMPs was comparable in samples pre-incubated with vehicle and epicatechin. The changes in CD42b-positive PMP levels in the small subpopulation were associated with a decrease in the median CD42b fluorescence signal from 85 ± 77 in samples incubated with vehicle to 45 ± 50 (*p* < 0.01), 61 ± 40 (*p* < 0.5) and 46 ± 45 (*p* < 0.5) in samples incubated with 1 µM, 10 µM and 100 µM epicatechin, respectively (Figure 2).

In all experimental conditions, incubation with epicatechin induced an imbalance in the small to large PMP ratio (%). After incubation with PBS, this ratio decreased from 9.4 ± 5.6 in samples incubated with vehicle to 8.3 ± 5.3 (*p* = 0.9), 6.8 ± 5.1 (*p* < 0.01) and 6.4 ± 4.5 (*p* < 0.05) in samples incubated with 1 µM, 10 µM and 100 µM epicatechin, respectively. After incubation with the calcium ionophore, this ratio decreased from 31.9 ± 15.6 in samples incubated with vehicle to 25.7 ± 14.8 (*p* < 0.01), 26.9 ± 16.1 (*p* < 0.05) and 24.9 ± 10.8 (*p* < 0.05) in samples incubated with 1 µM, 10 µM and 100 µM epicatechin, respectively. After PRP incubation with TRAP, the ratio dropped from 20.3 ± 32.8 in samples incubated with vehicle to 11.0 ± 8.2, 8.7 ± 8.2 and 6.4 ± 4.8 (*p* < 0.05) in samples incubated with 1 µM, 10 µM and 100 µM epicatechin, respectively.

### 3.2. Epicatechin Effect on Microparticle-Induced Coagulation

**Figure 3:**
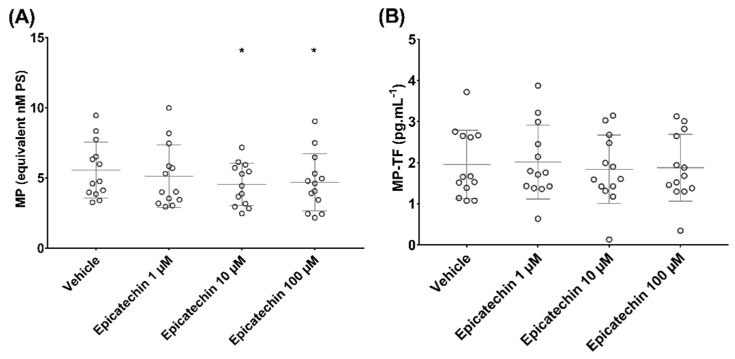
Effect of epicatechin on the concentration of microparticles (MPs) that express (**A**) phosphatidylserine (PS) and (**B**) tissue factor (TF). * Significant effect of intensity.

Epicatechin influenced microparticle concentration when their procoagulant activity in response to phospholipids was assessed using the Zymuphen^TM^ MP activity kit. PS-positive microparticle concentration (nM PS) decreased from 5.6 ± 2.0 in samples incubated with vehicle to 5.1 ± 2.2 (*p* = 0.5), 4.5 ± 1.5 (*p* < 0.05) and 4.7 ± 2.0 (*p* < 0.05) in samples incubated with 1, 10 and 100 µM epicatechin, respectively (Figure 3A).

No impact of epicatechin on TF-positive microparticle concentration (pg/mL) was observed when the procoagulant activity was induced by TF and assessed using the Zymuphen^TM^ MP-TF kit: 2.0 ± 0.8 in samples incubated with vehicle, and 2.0 ± 0.9 (*p* = 0.9), 1.8 ± 0.8 (*p* = 0.9) and 1.9 ± 0.8 (*p* = 0.9) in samples incubated with 1, 10 and 100 µM epicatechin, respectively (Figure 3B).

**Figure 4:**
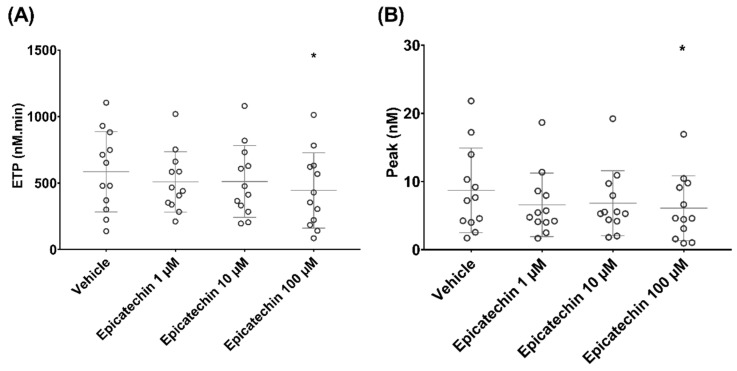
Effect of epicatechin on thrombin generation in microparticle-rich plasma. (**A**) Endogenous thrombin potential (ETP). (**B**) Thrombin peak. * Significant effect of intensity.

Epicatechin influenced TGA performed with MRP as source of phospholipids and TF. ETP (nM.min) decreased from 586 ± 302 in samples incubated with vehicle to 509 ± 226 (*p* = 0.3), 512 ± 270 (*p* = 0.3) and 445 ± 283 (*p* < 0.05) in samples incubated with 1, 10 and 100 µM epicatechin, respectively (Figure 4A). Thrombin peak (nM) also decreased from 9 ± 6 in samples incubated with vehicle to 7 ± 5 (*p* = 0.2), 7 ± 5 (*p* = 0.4) and 6 ± 5 (*p* < 0.05) in samples incubated with 1, 10 or 100 μM epicatechin, respectively (Figure 4B). Thrombin activation kinetics tended to change, but not significantly. Specifically, lag time and time to peak ranged from 40.2 ± 19.5 and 69 ± 32 in samples incubated with vehicle to 53.1 ± 25.2 and 88 ± 38 in samples incubated with 100 µM epicatechin, respectively.

**Figure 5:**
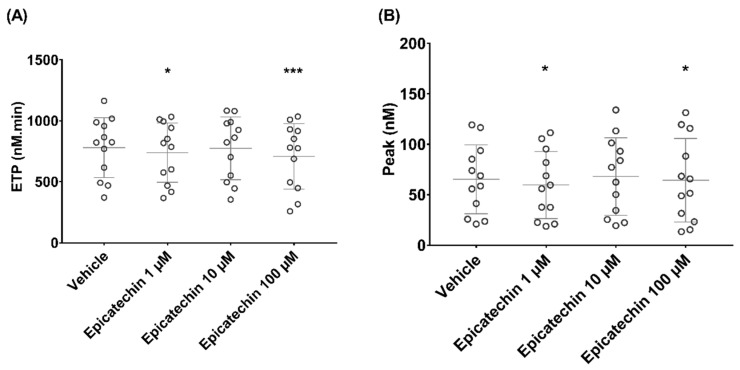
Effect of epicatechin on thrombin generation in microparticle-rich plasma after microparticle release stimulation by incubation with the calcium ionophore. (**A**) Endogenous thrombin potential (ETP). (**B**) Thrombin peak. *, *** Significant effect of intensity.

In calcium-stimulated PRP, ETP decreased from 779 ± 246 in samples incubated with vehicle to 739 ± 243 (*p* < 0.05), 774 ± 257 (*p* = 0.4) and 708 ± 268 (*p* < 0.001) in samples incubated with 1, 10 and 100 µM epicatechin, respectively (Figure 5A). Thrombin peak also decreased from 65 ± 34 in samples incubated with vehicle to 60 ± 33 (*p* < 0.05), 68 ± 33 (*p* = 0.6) and 64 ± 41 (*p* < 0.05) in samples incubated with 1, 10 and 100 μM epicatechin, respectively (Figure 5B). Thrombin activation kinetics were similar in all experimental conditions, with a lag time of about 15 min and a time to peak of about 22 min.

These data showed that 100 µM epicatechin can inhibit microparticle-mediated coagulation without affecting the lag time of thrombin generation

## 4. Discussion

**Figure 6:**
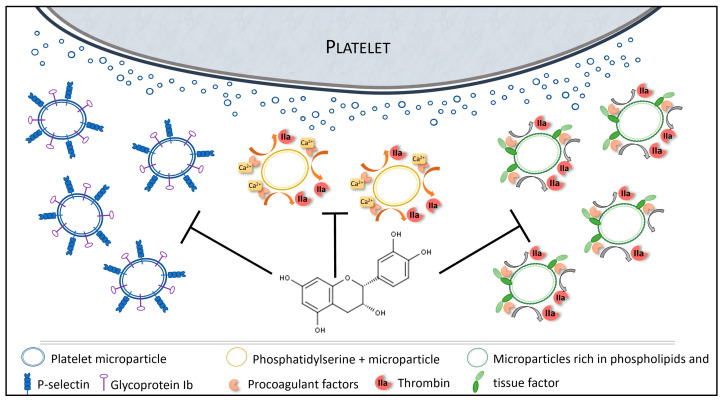
Overview of the impacts of epicatechin on microparticles.

There is strong evidence of the antiplatelet effects of dietary polyphenols, and it is suggested that polyphenols may have an impact on microparticles [42]. The present study investigated epicatechin role in microparticle formation and procoagulant potential that plays a key role in CVD (Figure 6) [43,44].

Our findings show that overall, epicatechin does not influence the concentration of PMPs, which were identified by labeling with the constitutive platelet receptor CD41a, even after platelet activation by agonists, such as TRAP or a calcium ionophore. However, some PMP sub-populations seem to be affected by epicatechin. Indeed, the percentage of total (small and large) P-selectin-expressing PMPs decreased, while PS expression was unchanged. The expression of GpIb at the PMP surface, particularly in small PMPs, was reduced already by incubation with 1 μM of epicatechin. Epicatechin also generated an imbalance between large and small GpIb-positive PMPs, following stimulation with the calcium ionophore, by increasing the large microparticle fraction.

There is evidence that microparticles smaller than 0.5 µm have specific properties, such as inhibition of the collagen/adenosine-diphosphate-mediated formation of platelet thrombi [45]. The epicatechin-induced decrease in GpIb-positive microparticles could impair their reactivity to thrombin and von Willebrand factor [46], and might have a direct impact on myocardial infarction pathogenesis [47]. Furthermore, epicatechin effect from 1 µM on P-selectin-expressing PMPs could have a beneficial effect on the risk of major adverse cardiovascular events after myocardial infarction [48,49], probably because of P-selectin role in thrombosis and in the recruitment of leukocytes in inflammation [50,51].

Microparticles are procoagulant factors due to their membrane that supports the coagulation enzymatic cascade. This property is reinforced by anionic phospholipids (e.g., PS) and TF, the main coagulation activator [52]. Here, we observed a functional impact of epicatechin on microparticle procoagulant role. Specifically, incubation with epicatechin reduced their phospholipid-mediated procoagulant activity (from 10 µM of epicatechin), but not the activity mediated by TF. Interestingly, TGA, which uses microparticles as phospholipid source and TF to trigger coagulation, is decreased by epicatechin. These data seem to demonstrate that epicatechin can inhibit microparticle-mediated coagulation by affecting the number and phenotype of the released microparticles, and/or the enzymatic reaction of coagulation.

Several studies have shown that initiation of thrombin generation is mainly supported by microparticles derived from monocytes, and not by PMPs [53,54]. However, PMPs contribute to clot propagation and to prothrombotic activities after initiation [55], Surprisingly, we found that PS-expressing PMPs were not modified by epicatechin. It was previously reported that epicatechin can inhibit thrombin activity [56,57]. This could explain the effect on the procoagulant activity mediated by phospholipids and on thrombin generation, and the absence of effect on the procoagulant action mediated by TF. Taken together, these data support epicatechin interest in CVD through its action on microparticles (PMP generation and size/properties) and on thrombin generation. Its capacity to modulate microparticles could contribute to its health protective effects [58].

This study has some limitations. Ottaviani et al. showed that among the stereoisomers of flavan-3-ol, (-)-epicatechin is the one with the highest bioavailability [59]. It would also be interesting to explore the effect of long-term in vivo exposure to lower concentrations of epicatechin, instead of short in vitro exposure to higher concentrations [60]. In vivo, epicatechin is present in the plasma as conjugated derivatives, resulting from phase II metabolism occurring after its intestinal absorption [61]. More studies are needed to thoroughly assess the bioactivity of epicatechin metabolites at physiologically relevant concentrations that we could not perform because they are not available yet. This is an in vitro study that must be completed by mechanistic investigations.

In conclusion, we demonstrated that epicatechin positively affects microparticle generation, phenotype and procoagulant properties, particularly PMPs. Given microparticle importance in CVD and the major complications of CVD, these data open new perspectives on how epicatechin can affect coagulation that deserve to be confirmed in in vivo studies.
